# It is not ‘business as usual’ for orthopaedic surgeons in May 2020– the Austrian-German-Swiss experience

**DOI:** 10.1186/s40634-020-00272-4

**Published:** 2020-08-08

**Authors:** M. C. Liebensteiner, I. Khosravi, M. T. Hirschmann, P. R. Heuberer, The Board of the AGA - Society of Arthroscopy and Joint-Surgery, Philipp HEUBERER, Philipp NIEMEYER, Helmut LILL, Christoph LAMPERT, Florian DIRISAMER, Sepp BRAUN, Tomas BUCHHORN, René E. L. ATTAL, Christian JUNG, Andreas Marc MÜLLER, Sven SCHEFFLER, Johannes ZELLNER, Peter ANGELE, M. Saffarini, M. Thaler

**Affiliations:** 1 Department of Orthopaedic Surgery, Medical University of Innsbruck, Innrain 52, 6020 Innsbruck, Austria; 2 Department of Orthopaedic Surgery and Traumatology, Kantonsspital Baselland, (Bruderholz, Liestal, Laufen), 4101 Bruderholz, Switzerland; 3 University of Basel, Basel, Switzerland; 4 Schulterzentrum Wien, HealthPi Medical Center, Vienna, Austria; 5 ReSurg SA, Nyon, Switzerland

**Keywords:** Coronavirus disease 2019, COVID-19, Severe acute respiratory syndrome coronavirus 2, SARS-CoV-2, Arthroscopy, Ligament reconstruction, Arthroplasty, Orthopaedic, Healthcare

## Abstract

**Purpose:**

To document the status-quo of orthopaedic health-care services as the COVID-19 pandemic recedes, and to determine the rate of resumption of orthopaedic surgery in the German-speaking countries in May 2020.

**Methods:**

A prospective online survey was sent out to 4234 surgeons of the AGA - Society of Arthroscopy and Joint-Surgery (Gesellschaft für Arthroskopie und Gelenkchirurgie, AGA). The survey was created using SurveyMonkey software and consisted of 23 questions relating to the reduction of orthopaedic services at the participating centres and the impact that the pandemic is having on each surgeon.

**Results:**

A total of 890 orthopaedic surgeons responded to the online survey. Approximately 90% of them experienced a reduction in their surgical caseload and patient contact. 38.7% stated that their institutions returned to providing diagnostic arthroscopies. 54.5% reported that they went back to performing anterior cruciate ligament reconstructions (ACLR), 62.6% were performing arthroscopic meniscus procedures, and 55.8% had resumed performing shoulder arthroscopy. Only 31.9% of the surgeons were able to perform elective total joint arthroplasty. 60% of the participants stated that they had suffered substantial financial loss due to the pandemic.

**Conclusion:**

A gradual resumption of orthopaedic health-care services was observed in May 2020. Typical orthopaedic surgical procedures like ACLR, shoulder arthroscopy and elective total joint arthroplasty were reported to be currently performed by 54%, 56% and 32% of surgeons, respectively. Despite signs of improvement, it appears that there is a prolonged curtailment of orthopaedic health-care at present in the middle of Europe.

## Introduction

The coronavirus disease 2019 (COVID-19) pandemic, caused by the severe acute respiratory syndrome coronavirus 2 (SARS-CoV-2), is challenging the medical community all over the world. Medical resources were reallocated to provide the best care to COVID-19 patients. As a consequence, orthopaedic daily practice has been affected significantly. Elective cases were postponed and urgent surgery required special attention for SARS-CoV-2 positivity [1, 2]. Those changes in orthopaedic health-care services have already been documented in the last months [3, 4].

Thaler et al. reported a massive reduction in hip and knee arthroplasty across Europe, based on a survey among 272 members of the European Hip Society and the European Knee Associates [4]. Of the respondent surgeons, more than 90% stated that their institutions no longer provided primary total joint arthroplasty. The same findings were reported for aseptic revision arthroplasty. Our own group conducted a similar online survey within the AGA - Society of Arthroscopy and Joint-Surgery - to investigate potential reductions in orthopaedic health-care services [3]. The online survey was conducted at the peak of the pandemic between 30 March 2020 and 5 April 2020. Of the 1399 respondent orthopaedic surgeons from Austria, Germany and Switzerland, only 50% stated that they were still performing rotator cuff repairs, and just 20% reported that they were performing anterior cruciate ligament reconstructions (ACLR). Likewise, only 30% of the respondents stated that they continue to provide arthroscopic meniscus surgery. 11.9% said that they were unable to follow-up patients that they had operated, and 35.1% confirmed that their patients still had access to outpatient physical therapy. Overall, the study reported a massive cutback in orthopaedic health-care services.

However, several countries have recently initiated a gradual resumption of orthopaedic procedures. In this context, several authors also published recommendations on how such a resumption should be carried out [2, 5, 6]. The present survey aimed to evaluate the situation of orthopaedic surgery for non-COVID-19 patients slightly after the peak of the pandemic while a gradual recovery of orthopaedic surgery was already initiated. We aimed to apply the same design and methods as the aforementioned study [3].

## Materials and methods

This was a prospective online survey of orthopaedic surgeons (expert opinion). All participants were members of the AGA - Society of Arthroscopy and Joint-Surgery (Gesellschaft für Arthroskopie und Gelenkchirurgie, AGA, www.aga-online.ch), currently Europe’s largest arthroscopy society. Of the 5339 members, 4234 are orthopaedic surgeons. Approval by an institutional review board was deemed unnecessary since the study did not involve patient data.

The survey was created using SurveyMonkey software (http://www.surveymonkey.com), an online data collection program, and comprised 23 questions relating to the following information: the background and surgical experience of the participant, the reduction of orthopaedic services at the participating centres and the impact of the pandemic on each individual surgeon. (see Appendix 1 for survey details).

A link to the survey was then sent by email to the above-mentioned 4234 orthopaedic surgeons on 1 May 2020. Every second day, those who had not yet responded received a reminder email, and the online survey was finally closed on 14 May 2020.

## Results

**Fig. 1:**
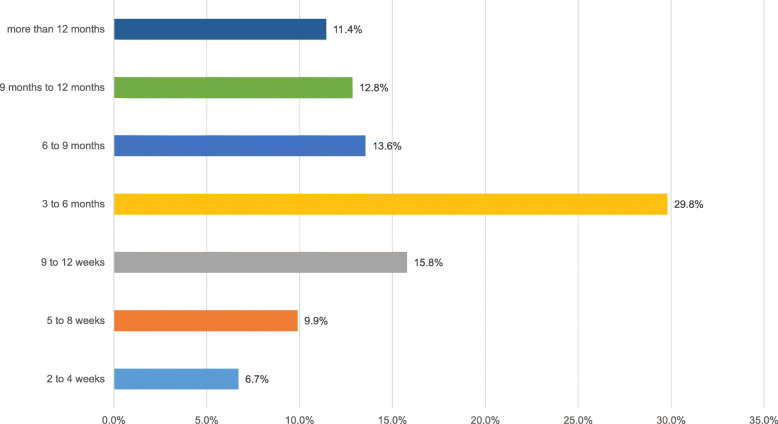
Surgeons’ answers to the question on how long they believe that the pandemic will impair their work

A total of 890 orthopaedic surgeons participated in the online survey; of whom 14.5% worked in Austria, 12.6% in Switzerland and 72.9% in Germany. 43.9% of the respondents reported having more than 20 years of professional experience (Table 1). 28.9% of the respondents reported financial losses of approximately 25%, 15.6% of the participants reported financial losses of 50% and 9.1% stated that they had approximately 75% losses. A 100% reduction of the business was reported by 0.6% of the surgeons. 24% of respondents estimated that the pandemic will affect their work for at least 9 months (Fig. 1).

**Table 1:** Distribution of the professional experience of the participants

Years of professional experience	%	Number of surgeons
1–3 years	4.2%	37
3–6 years	5.6%	50
6–10 years	10.7%	95
> 10 years	15.8%	141
> 15 years	19.8%	176
> 20 years	29.0%	258
> 30 years	13.6%	121
> 40 years	1.1%	10
> 50 years	0.2%	2

Regarding the provision of health-care services, the majority (approx. 75%) stated that their patients had to undergo COVID-19 screening by questionnaire and body temperature reading before receiving orthopaedic care. Those who screened positive were further tested for SARS-Cov-2. Approximately 90% of the surgeons reported substantial on-going reductions in their surgical caseload and patient contact. 38.7% confirmed that their institutions were still providing diagnostic arthroscopies (52.7% stopped, 8.6% stated that this procedure is generally not provided at their department). Regarding the resumption of more specific arthroscopic procedures, 54.5% of the surgeons surveyed reported that they were again performing ACLR (38.9% still stopped, 6.7% stated that the procedure is generally not provided). 62.6% were performing arthroscopic meniscus procedures (34.5% still stopped, 2.9% stated that the procedure is generally not provided).

**Fig. 2:**
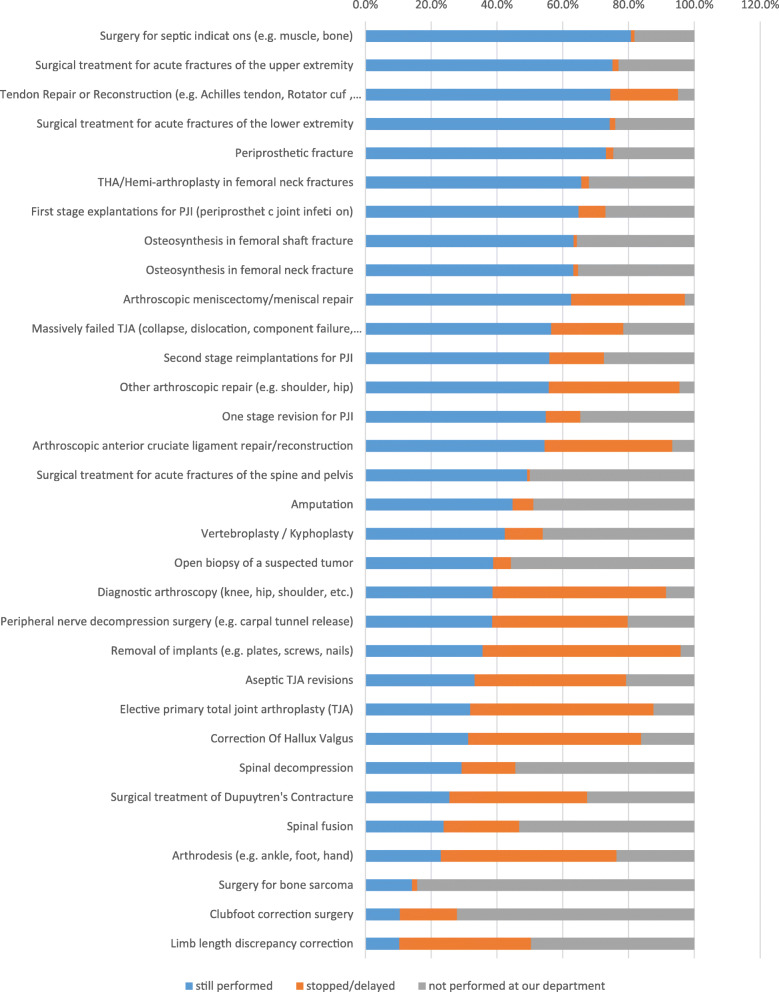
Statements of the 890 study participants on whether specific orthopaedic surgical procedures are provided at their institutions. PJI: periprosthetic joint infection, TJA: total joint arthroplasty, THA: total hip arthroplasty

**Fig. 3:**
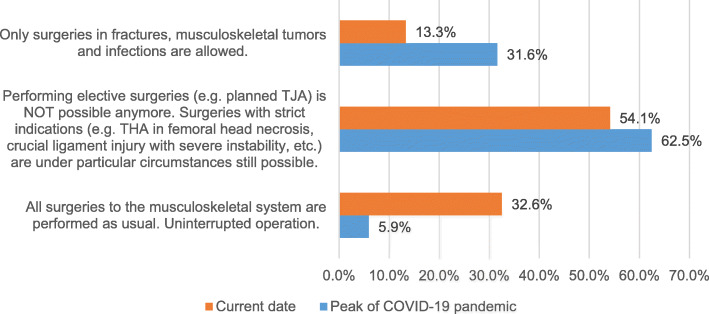
Stages of escalation caused by the pandemic as rated by the survey respondents. The participants were asked about the status of their institutions. Blue bars represent the answers provided at the peak of the pandemic [3]. Orange bars represent the answers of the current study in May 2020

There were major disruptions in elective total joint arthroplasty, with only 31.9% of the surgeons being able to provide such surgery (55.7% still stopped, 12.4% stated that the procedure is generally not provided). The numbers of aseptic revisions of total joint arthroplasty were almost identical (Table 2, Fig. 2). The respondents rated the stages of escalation to be lighter at present than at the peak of the pandemic (Fig. 3).

**Table 2:** Statements of the 890 study participants on whether specific orthopaedic surgical procedures are provided at their institutions. PJI: periprosthetic joint infection, TJA: total joint arthroplasty

	Status		
	performed	stopped/delayed	not provided at the department
Surgery for septic indications (e.g. muscle, bone)	80.7%	1.1%	18.2%
Surgical treatment for acute fractures of the upper extremity	75.2%	1.8%	23.1%
Surgical treatment for acute fractures of the lower extremity	74.3%	1.7%	24.0%
Periprosthetic fracture	73.1%	2.3%	24.6%
Hip arthroplasty in femoral neck fractures	65.7%	2.3%	32.1%
Osteosynthesis in femoral neck fracture	63.2%	1.4%	35.3%
Osteosynthesis in femoral shaft fracture	63.3%	1.1%	35.7%
First stage explantations for PJI	64.8%	8.2%	27.0%
Tendon Repair or Reconstruction	74.5%	20.6%	5.0%
Surgical treatment for acute fractures of the spine and pelvis	49.2%	0.8%	50.0%
One stage revision for PJI	54.8%	10.5%	34.7%
Second stage reimplantations for PJI	55.9%	16.6%	27.5%
Massively failed TJA (collapse, dislocation, component failure, imminent dislocation)	56.4%	22.0%	21.6%
Amputation	44.7%	6.4%	48.9%
Arthroscopic meniscectomy/meniscal repair	62.6%	34.5%	2.9%
Open biopsy of a suspected tumor	38.9%	5.4%	55.8%
Vertebroplasty/Kyphoplasty	42.4%	11.5%	46.1%
Arthroscopic reconstructions at shoulder/hip	55.8%	39.7%	4.5%
Arthroscopic anterior cruciate ligament reconstruction	54.5%	38.9%	6.7%
Spinal decompression	29.3%	16.4%	54.4%
Peripheral nerve decompression surgery (e.g. carpal tunnel release)	38.6%	41.2%	20.2%
Surgery for bone sarcoma	14.2%	1.6%	84.2%
Aseptic TJA revisions	33.2%	46.0%	20.7%
Diagnostic arthroscopy (knee, hip, shoulder, etc.)	38.7%	52.7%	8.6%
Removal of implants (e.g. plates, screws, nails)	35.6%	60.2%	4.2%
Spinal fusion	23.8%	23.0%	53.2%
Correction Of Hallux Valgus	31.3%	52.6%	16.2%
Surgical treatment of Dupuytren’s Contracture	25.6%	41.8%	32.6%
Elective primary TJA	31.9%	55.7%	12.4%
Clubfoot correction surgery	10.5%	17.4%	72.1%
Arthrodesis (e.g. ankle, foot, hand)	22.9%	53.4%	23.7%
Limb length discrepancy correction	10.3%	40.0%	49.6%

About 50% of the surgeons surveyed reported that conferences were still compromised (cancelled, reduced staff, videoconference only). Only 55.8% felt that their institutions provided them with sufficient personal protective equipment (PPE). 78.6% of the survey participants stated that they had resumed carrying out postoperative check-ups on their patients. 54.7% of respondents confirmed the availability of outpatient physical therapy. However, only 35.2% reported that they could send their patients to rehabilitation centres postoperatively (Table 3).

**Table 3:** Statements of the surgeons when asked on whether physical therapy is available for their patients

Physical Therapy is not available at all	2.6%
Physical therapists are treating my postoperative patients at the ward	69.8%
Physical Therapy is provided by Rehabilitation Centers	35.2%
Physical Therapy is available for outpatients in physical therapist’s practices	54.7%
Physical Therapy is available for my patients, but ressources are insufficient	26.4%
Miscellaneous	2.4%

## Discussion

The most important finding of our study is that, in May 2020, the COVID-19 pandemic is still drastically curtailing the availability of orthopaedic services in the German-speaking countries of Europe, with approximately 90% of the 890 participants still reporting substantial reductions in their surgical caseload and patient contact. Substantial cutbacks in typical arthroscopic procedures persist, with only 38.7% of the respondents being able to perform diagnostic arthroscopy (52.7% stopped, 8.6% stated that this is generally not provided). 54.5% stated that they were currently able to offer ACLR (38.9% still stopped and 6.7% generally not provide that procedure).

Compared to our previous group survey, conducted at the peak of the COVID-19 pandemic [3], the present study found that there was a gradual return towards normality. However, in May 2020 patients with orthopaedic disorders are still not receiving the full range of services. While at the peak of the pandemic one quarter of the surgeons were able to offer ACLR, the capacity has now approximately doubled (54%). This is also true for the provision of diagnostic arthroscopy where the rate has improved from 11% to 38%, whereas for elective total joint arthroplasty the rate has improved from 6% to 30%. Apart from the availability of surgical procedures, over the same period, the availability of outpatient physical therapy was reported to have improved from 35% to approximately 55% of the participants. So overall, despite some gradual improvement, it seems that there is still a drastic curtailment in the provision of orthopaedic health-care now happening in the German-speaking countries of Europe.

Recently, recommendations have been published on how to provide orthopaedic health-care as the COVID-19 pandemic recedes [5, 7, 8]. Mouton et al. reported that elective procedures should only be carried out in absolutely COVID-free facilities and that hospital stay should be as short as possible [8]. They further suggested careful checking of the applicants according to COVID infection status/exposure, age, health status/risk factors, socio-professional status and surgical indication. However, we are not aware of studies that have actually documented that process, nor if and how orthopaedic health-care will return to normal.

The above-mentioned cutback in orthopaedic health-care should not be viewed as merely deferring procedures without any consequences. It is well known that pain and disability rapidly progress during the waiting period in cases that require knee or hip arthroplasty [9, 10]. Glazebrook et al. investigated the quality of life of patients with hip and ankle osteoarthritis while waiting for surgery [11]. Quality of life (Short-Form 36) was found to be approximately two standard deviations below normal population scores. It is also well known that patients waiting for hip or knee arthroplasty are often prescribed opioids, leading to increased postoperative opioid tolerance, potential opioid addiction, increased perioperative complication rates and overall costs [12–14].

It may also be speculated that the delay in orthopaedic surgical procedures may even lead to poorer postoperative outcomes. With this in view, Mossmayer et al. prospectively followed 103 patients with rotator cuff tears over ten years and found that those with delayed repair had significantly poorer results than those who underwent early repair - an effect that worsened with every passing year [15]. The same was also studied in patients waiting for ACLR [16]. The authors stated that waiting time was significantly associated with secondary meniscus and cartilage lesions. Negative effects of longer waiting times on clinical postoperative outcomes were also reported for lumbar spine surgery [17] and total knee arthroplasty [18, 19].

Beyond the issue of impaired medical services for patients there is also a substantial cost concern. Only 40% of participants stated that they suffered no financial loss due to the pandemic. The rest were significantly affected by the imposed restrictions. As with other businesses, it is not yet clear whether some orthopaedic surgeons will have to close their practice due to bankruptcy, thereby potentially further contributing to reduced orthopaedic care and longer waiting lists.

The following limitations must be acknowledged. First, the findings from Austria, Germany and Switzerland cannot be generalized to all neighbouring countries. Second, the survey participants were members of the ‘AGA - Society of Arthroscopy and Joint-Surgery’ with a special interest in sports orthopaedics and joint preserving surgery. Therefore, surgeons performing high volumes of total joint arthroplasty may have been underrepresented in our survey.

One of the strengths of this study is that it is the first to actually document the process of determining if and how orthopaedic health-care will return to normal after the peak of the COVID-19 pandemic. Another strong point is the high participation number (890) of orthopaedic surgeons from the German-speaking countries. Over 100 million people live in Austria, Germany and Switzerland, representing approximately one-seventh of Europe’s population. The survey should therefore be regarded as providing robust information on the status of orthopaedic health-care as the Covid-19 epidemic recedes.

## Conclusions

A gradual resumption of orthopaedic health-care services was documented for May 2020. Typical orthopaedic surgical procedures such as ACLR, shoulder arthroscopies and elective total joint arthroplasty were reported as being currently carried out by 54%, 56% and 32% of surgeons, respectively. Despite a gradual improvement, it seems that there will be a prolonged curtailment of orthopaedic health-care in the middle of Europe.

## Supplementary information

**Additional file 1: Appendix 1.**. AGA COVID-19 Orthopedic practice Survey
