# Supplementary material for: It is not ‘business as usual’ for orthopaedic surgeons in May 2020– the Austrian-German-Swiss experience
Source: J Exp Orthop. 2020 Aug 8;7:61. doi: 10.1186/s40634-020-00272-4 (PMC7414630; doi:10.1186/s40634-020-00272-4)
Supplement: Supplementary file 1 — Additional file 1: Appendix 1.. AGA COVID-19 Orthopedic practice Survey [file 40634_2020_272_MOESM1_ESM.pdf]

AGA COVID-19 Orthopedic practice Survey

Thank you for participating in this survey.

We would like to ask you to kindly take approximately 8 minutes of your time andcomplete an online survey regarding the COVID-19 pandemic and how it affects yourpatients and your practice.

All data and questions will be absolutely anonymous. All answered questions will be savedso that you will be able to return to your latest question and go on with the survey fromthat question.

1. In which country are you currently working?

Country ____

State ____

2. What is your orthopedic speciality? (Mark all that apply)

- ☐ Shoulder
- ☐ Elbow
- ☐ Hand and Wrist
- ☐ Knee
- ☐ Hip
- ☐ Spine
- ☐ Foot and Ankle
- ☐ Musculoskeletal oncology
- ☐ Adult - Knee reconstruction
- ☐ Adult - Shoulder reconstruction
- ☐ Adult - Hip reconstruction
- ☐ Sports-medicine
- ☐ Pediatric orthopedics
- ☐ Traumatology
- ☐ General orthopedics
- ☐ Resident/ Fellow - not specified yet
- ☐ None of the above or other (please specify)

____

3. In which type of environment are you working? (Mark all that apply)

- ☐ Academic medical center
- ☐ Public hospital
- ☐ Private hospital
- ☐ Private practice

4. How many years have you been practicing?

- ◯ 1-3
- ◯ 3-6
- ◯ 6-10
- ◯ >10
- ◯ >20
- ◯ >30
- ◯ >40
- ◯ >50

5. Have you been quarantined?

- ◯ yes
- ◯ no

6. Have you suffered financial loss?

- ◯ No
- ◯ Yes, 25% financial loss
- ◯ Yes, 50% financial loss
- ◯ Yes, 75% financial loss
- ◯ Yes, 100% financial loss
- ◯ Other (please specify)

7. Do you fear infecting your friends or family and what is your approach forprevention? (Mark all that apply)

- ☐ Yes, I don't go home anymore (stay at the hospital, hotel, second apartement, etc.)
- ☐ Yes, I wash and disinfect my hands more often than usual
- ☐ Yes, I change my clothes in the hospital more often
- ☐ Yes, I try to keep a distance to my family at home
- ☐ Yes, I don't stay in the same room with other members of my family anymore
- ☐ Yes, I disinfect surfaces in my home after I touch them
- ☐ Other (please specify)
- ☐ Yes, I wear a surgical mask/other protection at home
- ☐ Yes, I avoid close physical contact with my family members
- ☐ Yes, I am more careful at work than usual
- ☐ Yes, I took off from work
- ☐ No, I don't care at all
- ☐ No, I haven't thought about this situation

8. What specific effects has the COVID-19 pandemic had on your department?(Mark all that apply)

- ☐ No changes at the department
- ☐ All surgeries have been stoppedstopped
- ☐ The department has **stopped** *elective inpatient* surgery
- ☐ The department has **stopped** elective *outpatient* surgery
- ☐ The hospital has **selectively restricted** *inpatient* elective surgery
- ☐ The hospital has **selectively restricted** *outpatient* elective surgery

11. Are the following procedures currently being performed currently being performed at your departement?

	yes	stopped	delayed	not provided at our department
"elective" Primary total joint arthroplasty (TJA)				
First stage explantations for PJI (periprosthetic joint infection)				
Second stage re-implantations for PJI				
One stage revision for PJI				
Aseptic TJA revisions				
Massively failed TJA (collapse, dislocation,component failure, imminent dislocation)				
Periprosthetic fracture				
Removal of implants (e.g. plates, screws,nails)				
Osteosynthesis in femoral neck fracture				
Osteosynthesis in femoral shaft fracture				
THA/hemi-arthroplasty for femoral neckfractures				
Surgery for septic indications (e.g. muscle,bone)				
Limb length discrepancy correction				
Arthrodesis (e.g. ankle, foot, hand)				
Surgery for bone sarcoma				
Diagnostic arthroscopy (knee, hip, shoulder,etc.)				
Spinal fusion				
Spinal decompression				
Vertebroplasty /kyphoplasty				
Peripheral nerve decompression surgery (e.g. carpal tunnelrelease)				

Surgical treatment of Dupuytren's contracture	◯	◯	◯	◯
Arthroscopic anterior cruciate ligament repair/ reconstructionn)	◯	◯	◯	◯
Arthroscopic meniscectomy/ meniscal repair	◯	◯	◯	◯
Other arthroscopic repair (e.g. shoulder,hip)	◯	◯	◯	◯
Surgical treatment for acute fractures of the lower extremity	◯	◯	◯	◯
Surgical treatment for acute fractures of the upper extremity	◯	◯	◯	◯
Surgical treatment for acute fractures of the spine and pelvis	◯	◯	◯	◯
Clubfoot correction surgery	◯	◯	◯	◯
Open biopsy of a suspected tumor	◯	◯	◯	◯
Correction of hallux valgus	◯	◯	◯	◯
Tendon repair or reconstruction (e.g.Achilles tendon, rotator cuff, etc.)	◯	◯	◯	◯
Amputation	◯	◯	◯	◯

12. In which stage is your department which stage is your department at this timeat this time?

◯ All surgeries to the musculoskeletal system are being performed as usual. Uninterrupted operation.

◯ Performing elective surgeries (e.g. planned TJA) is **NOT** possible anymore. Surgeries with strict indications (e.g. THA in femoral head necrosis, crucial ligament injury with severe instability, etc.) are still possible under special circumstances.

◯ Only surgeries for fractures, musculoskeletal tumors and infections are allowed.

13. Has there been any specific COVID-19 training for your surgical staff?

◯ yes

◯ no

14. Has there been a positive COVID-19 test result (infection proved)? (Mark allthat apply)

- ☐ Patient in my hospital
- ☐ Patient in my department
- ☐ Health care professional in my hospital
- ☐ Health care professional in my department
- ☐ Other staff in my hospital
- ☐ Other staff in my department
- ☐ None of the above

15. Have there been any disruptions related to the pandemic? (Mark all thatapply)

- ☐ Staff disruptions
- ☐ Supply disruptions
- ☐ Missing regular intensive care units
- ☐ Missing regular intermediate care units
- ☐ Other (please specify)
- ☐ Missing regular inpatient beds
- ☐ Missing COVID-19 intensive care units
- ☐ Missing COVID-19 intermediate care units
- ☐ Missing COVID-19 inpatient beds

16. With regard to your regular meetings, is there any difference due to theCOVID-19 pandemic?

- ○ No difference at my department
- ○ Reduced staff at the meetings
- ○ No meetings anymore
- ○ Other (please specify)
- ○ **ALL** staff members participate at the meetings but keep a distance to eachother
- ○ **ALL** staff members participate at the meetings but wear protection (masks,coats etc.)
- ○ Meetings are held exclusively online via videoconference

17. Has protective clothing been provided to you? (Mark all that apply)

- ☐ Sufficient amount provided by the hospital
- ☐ No, I had to provide protective clothing myself
- ☐ I work without protective clothing
- ☐ Sufficient amount provided by the General medical council
- ☐ Insufficient amount provided by the General medical council
- ☐ Other (please specify)

18. What are your clinic's approaches to preventing sick leave due to COVID-19 pandemic? (Mark all that apply)

- [ ] Staff is separated into groups
- [ ] Personal protection (masks, coats,gloves etc.)
- [ ] Reduced staff appearance at meetings
- [ ] Remote working from home (scientificresearch, webinars, telemedicine etc.)
- [ ] Altered rotations in staff appearance
- [ ] No prevention

19. Do you offer patient care via technologies like telemedicine? (Mark all thatapply)

- [ ] Videoconference (Skype, Zoom, etc.)
- [ ] Telephone
- [ ] web based telemedicine (FaceTime, GoogleChat etc.)
- [ ] EHR/EMR (electronic healthrecord/ electronic medical record)
- [ ] None
- [ ] Other (please specify)

20. How long do you think the COVID-19 pandemic will affect your clinicalroutine/your surgical schedule?

- ○ 2 to 4 weeks
- ○ 5 to 8 weeks
- ○ 9 to 12 weeks
- ○ 3 to 6 months
- ○ 6 to 9 months
- ○ 9 to 12 months
- ○ more than 12 months

21. What impact has the COVID-19 pandemic had on you? (Mark all that apply)

- [ ] I am working as usual
- [ ] I am effectively not involved in any surgical activity due to institutional or self-imposed deferral of elective surgery, but am more focused on scientific and administrative work.
- [ ] I am not working due to personal illness, COVID-19 exposure, or post-travel quarantine
- [ ] Other (please specify)

9. What specific effect has the COVID-19 pandemic had on your outpatientclinic?

- ○ **ALL** patients are being tested for SARS-CoV-2 prior to orthopedic clinical examination
- ○ **ALL** patients are being screened for SARS-CoV-2 (e.g. having bodytemperature taken, answering a questionnaire, etc.) prior to orthopedicclinical examination
- ○ Patients with positivesymptoms/positive screening questionsare being tested for SARS-CoV-2
- ○ Other (please specify)
- ○ No changes at our outpatient clinic (yet)
- ○ Only patients with acute orthopedicsymptoms (fracture, infection, tumor eg.bone sarcoma) are allowed at ouroutpatient clinic

10. How has the COVID-19 pandemic affected YOUR practice as an orthopedic surgeon? (Mark all that apply)

- ☐ No impact
- ☐ My volume of performed surgery is reduced
- ☐ Discussing the delay due to the pandemic with patients
- ☐ No Training or teaching (students,residents, fellows) due to the pandemic
- ☐ More non-surgical orthopedic clinicalcare is being performed
- ☐ More administrative work is being done
- ☐ Orthopedic surgeons are assigned moreoften to non-orthopedic patient care due to the pandemic
- ☐ The patients occurence is reduced

22. Do you still perform follow up investigations? (Mark all that apply)

- [ ] Yes, clinical follow up
- [ ] Yes, radiological follow up
- [ ] Yes, I take the sutures out myself
- [ ] Other (please specify)
- [ ] Yes, but I follow up only high risk patients (e.g. complex revisions, septic, etc.)
- [ ] No, the sutures are taken out by someone else (e.g. general physician/family doctor)
- [ ] No, the patients are not followed up anymore

23. Is any physical therapy or rehabilitation offered for already discharged TJA patients? (Mark all that apply)

- [ ] Professional physiotherapy or rehabilitation is no longer available
- [ ] Yes, physiotherapy is available during the in hospital stay after surgery.
- [ ] Yes, physiotherapy is available in rehabilitation centers
- [ ] Other (please specify)
- [ ] Yes, physiotherapy is available in private practice
- [ ] Yes physiotherapy is possible in principle, but ressources are limited

24. Do you want to leave any comment?
